# The delivery of Ask-Advise-Connect for smoking cessation in Dutch general practice during the COVID-19 pandemic: results of a pre-post implementation study

**DOI:** 10.1186/s12913-023-09692-1

**Published:** 2023-06-19

**Authors:** Naomi A. van Westen-Lagerweij, Marc C. Willemsen, Esther A. Croes, Niels H. Chavannes, Eline Meijer

**Affiliations:** 1 The Netherlands Expertise Centre for Tobacco Control, Trimbos Institute, PO Box 725, 3500 AS Utrecht, The Netherlands; 2 Department of Health Promotion, Maastricht University, PO Box 616, 6200 MD Maastricht, The Netherlands; 3 Public Health and Primary Care, Leiden University Medical Center, PO Box 9600, 2300 RC Leiden, The Netherlands; 4 National eHealth Living Lab, PO Box 9600, 2300 RC Leiden, The Netherlands

**Keywords:** Ask-Advise-Connect, Implementation, General practice, COVID-19 pandemic

## Abstract

**Background:**

The Ask-Advise-Connect approach can help primary care providers to increase the number of smokers that attempt to quit smoking and enrol into cessation counselling. The approach has not yet been implemented in general practice in the Netherlands. The aim of this study was to investigate the influence of a comprehensive implementation strategy on the delivery of Ask-Advise-Connect for smoking cessation within Dutch general practice during the COVID-19 pandemic.

**Methods:**

A pre-post study was conducted between late 2020 and early 2022, and included 106 Dutch primary care providers (GPs, practice nurses and doctor’s assistants). Participation lasted nine months: during the first three months participants delivered smoking cessation care as usual (pre-intervention); the implementation strategy came into effect after three months and participants were followed up for another six months (post-intervention). The implementation strategy consisted of two meetings in which participants were educated about Ask-Advise-Connect, made agreements on the implementation of Ask-Advise-Connect and reflected on these agreements. Participants also received online educational materials and a desk card as reminder. The changes in the proportions of ‘Ask’ and ‘Advise’ over time were modelled using linear mixed effects models. A descriptive analysis was conducted with regard to referrals to cessation counselling.

**Results:**

Participants provided consultations to 29,112 patients (both smokers and non-smokers). Results of the linear mixed effects model show that the proportion of patients that were asked about smoking (‘Ask’) significantly decreased in the first three months (pre-intervention), but slightly increased again after the implementation strategy came into effect (post-intervention). No significant change over time was found with regard to the proportion of patients advised to quit smoking (‘Advise’). Descriptive statistics suggested that more participants proactively (vs. passively) referred patients to cessation counselling post-intervention (‘Connect’).

**Conclusions:**

The findings indicate that a comprehensive implementation strategy can support primary care providers in offering smoking cessation care to patients, even under stressful COVID-19 conditions. Additional implementation efforts are needed to increase the proportion of patients that receive a quit advice and proactive referral.

**Supplementary Information:**

The online version contains supplementary material available at 10.1186/s12913-023-09692-1.

## Background

Primary care practice, or general practice, is an important setting for promoting tobacco cessation and supporting smokers in their endeavour to quit smoking [1]. The World Health Organization and most national clinical guidelines recommend that primary care providers document the smoking status of patients and offer advice and support to quit smoking to patients who smoke [1, 2]. A brief advice from a physician to quit smoking can increase quit rates by as much as 60% [3]. In addition, evidence suggests that the provision of behavioural counselling, pharmacotherapy, and tailored printed materials within the primary care setting contribute to more people who successfully quit smoking [4].

Previous research has shown that primary care providers in the Netherlands do not routinely implement the clinical guidelines for smoking cessation care in practice [5–7]. Time constraints, (expectations of) low motivation to quit among patients, and the assumed sensitivity of the subject are important barriers which prevent primary care providers from discussing smoking cessation and offering support [6–8]. This is unfortunate as primary care providers can play an important role in stimulating quit attempts and the use of professional support (i.e., behavioural counselling and pharmacotherapy) [3, 9]. Currently, the majority of European smokers, including those in the Netherlands, have not attempted to quit smoking in the last 12 months [10]. In addition, the majority does not make use of smoking cessation support during a quit attempt [10]. Around 95% of smokers who try to quit smoking without any professional support relapse within one year [11]. Increasing the uptake of smoking cessation support is therefore necessary to increase the number of smokers who successfully achieve abstinence.

In the Netherlands, the general practitioner (GP) is the most consulted healthcare professional, with over two-thirds of Dutch smokers consulting their GP every year [9]. The Dutch clinical guideline for smoking cessation follows the 5A approach, which recommends that GPs ask patients about tobacco use, advise smokers to quit smoking, and assess the willingness to quit among smokers [12]. Only smokers who are motivated to quit are offered assistance; preferably behavioural counselling [12]. For patients who smoke more than 10 cigarettes a day a combination of counselling and pharmacotherapy (nicotine replacement therapy or medication) is most effective and therefore recommended. Finally, follow-up is arranged for those who accept support.

Typical for the Dutch context is that smokers who accept support are usually referred to the practice nurse (PN) for behavioural counselling. Most Dutch general practices have such a PN [13, 14]. However, GPs may also decide to refer patients to counselling outside general practice, for example if the practice is faced with a high workload or if patients want or need a specific type of counselling which is not offered within practice, such as group counselling or specialized addiction care [15]. Counselling outside general practice is typically reimbursed in the Netherlands, as long as the counselling is evidence-based.

Considering the barriers which primary care providers experience in implementing the guidelines for smoking cessation care [6–8], alternatives to the 5A approach have been proposed which may offer a more feasible and quicker way of providing smoking cessation care, such as the Ask-Advise-Refer (AAR) approach. This approach limits the tasks of the GP and PN to asking, advising and arranging follow-up [16]. There is some evidence to suggest that leaving out the assessment of motivation and offering support to all smokers, results in more quit attempts [17].

Another effective approach is the Ask-Advise-Connect (AAC) method, which includes asking patients about tobacco use, advising all smokers to quit smoking, offering evidence-based support to all smokers, and proactively referring smokers to a counsellor [19]. Proactively referring smokers (i.e., ensuring that a patient is directly connected to a counsellor) results in higher enrolment rates compared to passively referring smokers as is done in the AAR approach (i.e., instructing patients to contact a counsellor themselves) [18]. A proactive referral can, for example, be provided by forwarding the contact details of the patient to a counsellor who in turn contacts the patient, or by directly scheduling an appointment for the patient with a counsellor. Considering the low quit attempt rates and the low uptake of smoking cessation counselling among Dutch smokers [9], AAC may be a promising approach to ensure that more smokers attempt to quit smoking and enrol into counselling. AAC has not yet been implemented in Dutch general practice.

Implementing new evidence-based approaches or guidelines in healthcare practice can be challenging, as different barriers may prevent primary care providers from translating guidelines into daily practice [6–8]. In addition, the ongoing COVID-19 pandemic poses new organisational challenges for general practices in the delivery of care, further complicating the translation of guidelines into practice. A comprehensive set of strategies aimed at enhancing the adoption and implementation of evidence-based guidelines may be necessary to successfully implement AAC in Dutch general practice, especially during COVID-19 times [19]. The current study investigated the influence of a comprehensive implementation strategy on the delivery of AAC for smoking cessation within Dutch general practice during the COVID-19 pandemic. We used several strategies which are known to be effective, including educating primary care providers about AAC, facilitating a collaboration in which primary care providers make agreements and reflect on the implementation of AAC, reminding primary care providers to use the new approach, and connecting primary care providers to counsellors outside the practice whom they can refer patients to [19, 20].

## Methods

### Design and intervention

From late 2020 to early 2022, we conducted a pre-post study among primary care providers in the Netherlands. We considered Pharmaceutical Therapeutic Audit Meeting (PTAM) groups (‘FTO’ groups in Dutch) to be a suitable structure for implementing the AAC method. In the Netherlands, most GPs participate in a PTAM group. A PTAM group is a local collaboration with an average of 12 primary care providers (i.e., GPs and pharmacists) per group. Members meet several times per year to discuss and agree on the implementation of clinical guidelines around various topics. Members receive accreditation points for participation.

Before the start of this study, we conducted focus groups with primary care providers to determine which factors may influence the delivery of AAC within general practice [15]. Based on the results and on effective strategies described in literature [21, 22], we developed a comprehensive implementation strategy which consisted of different elements. See Table 1 for an overview of these elements.

**Table 1:** Elements of the Ask-Advise-Connect implementation strategy

**Element**	**Description**	**Corresponding strategy from literature **[19, 20]	**Definition of strategy**
First PTAM	During the first PTAM^a^ (either on location or online), participants learned about the AAC method and made agreements about the implementation of the new method in practice (the agreements specified when, how and by whom AAC would be delivered in practice). Participants were also informed about different options for smoking cessation counselling and, if possible, introduced to a local counsellor outside the practice. The first PTAM was facilitated by a trained employee of the Dutch Institute for Rational Use of Medicine	Conduct educational meetings	“Hold meetings targeted toward different stakeholder groups to teach them about the clinical innovation.”
		Create a learning collaborative	“Facilitate the formation of groups of providers or provider organizations and foster a collaborative learning environment to improve implementation of the clinical innovation.”
		Engage community resources	“Connect practices and their patients to community resources outside the practice.”
Desk card	During the first PTAM, participants received a desk card which describes the AAC method (see Fig. 1)	Remind clinician	“Develop reminder systems designed to help clinicians to recall information and/or prompt them to use the clinical innovation.”
E-toolkit	After the first PTAM, participants received access to an online toolkit in which more information can be found about the AAC method	Distribute educational materials	“Distribute educational materials (including guidelines, manuals, and toolkits) in person, by mail, and/or electronically.”
Second PTAM	Three months after the first PTAM, a second meeting was organized in which participants reflected on the previously made agreements and discussed best practices and possible solutions to encountered barriers. Aggregated data on Ask and Advise of T1-T3 versus T3-T6 was presented, except for in one PTAM group where not enough data was collected on Ask and Advise. The second PTAM was facilitated by one of the study researchers	Organize clinician implementation team meetings	“Develop and support teams of clinicians who are implementing the innovation and give them protected time to reflect on the implementation effort, share lessons learned, and support one another’s learning.”
		Audit and provide feedback	“Collect and summarize clinical performance data over a specified time period and give it to clinicians and administrators to monitor, evaluate, and modify provider behaviour.”
Document ‘tips for barriers’	After the second PTAM, participants received an online document with an overview of the most frequently mentioned barriers and tips on how to overcome these barriers	Distribute educational materials	“Distribute educational materials (including guidelines, manuals, and toolkits) in person, by mail, and/or electronically.”

^a^*PTAM* Pharmaceutical Therapeutic Audit Meeting

**Fig. 1:**
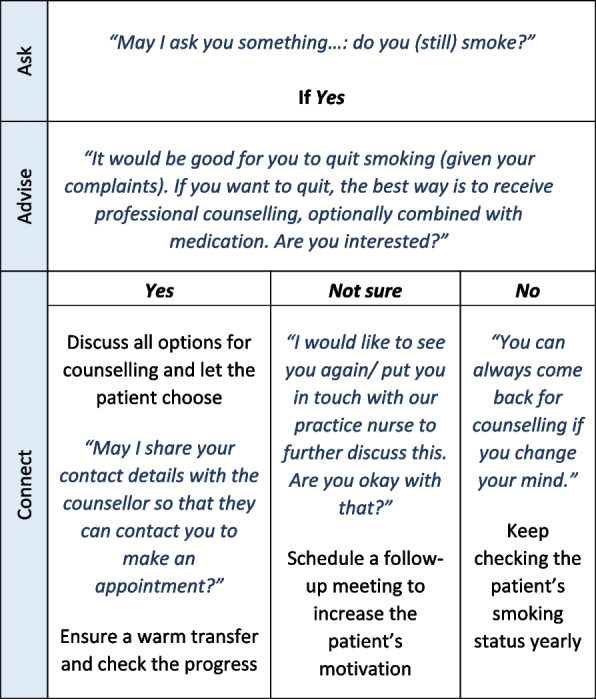
Ask-Advise-Connect desk card

With regard to the AAC method, we included the components as described in the literature by Vidrine et al. [18] (i.e., asking patients about tobacco use, advising all smokers to quit smoking, offering evidence-based support to all smokers, and proactively referring smokers to cessation support). We also extended the quit advice to include information on the best way to quit, and based on the patient’s interest in counselling we distinguished between ‘interested’, ‘not sure’, and ‘not interested’ with corresponding follow-up answers (see Fig. 1).

**Fig. 2:**
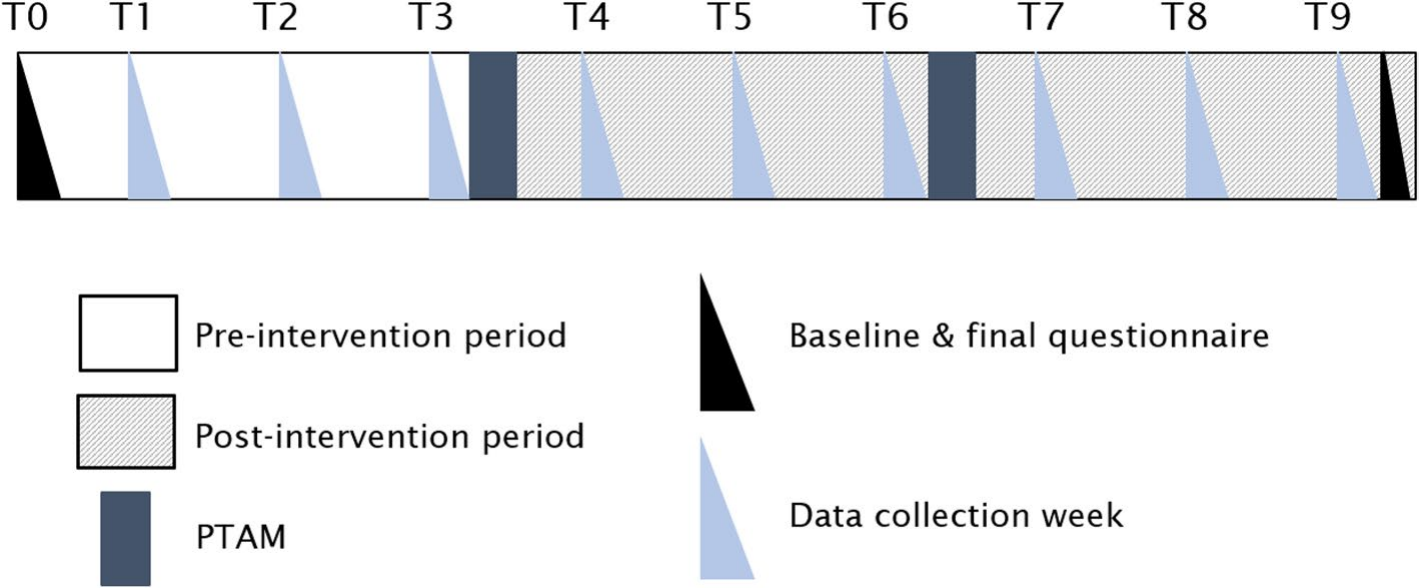
Study timeline

The duration of study participation was nine months. During the first three months participants delivered smoking cessation care as usual. The AAC method was introduced after three months of participation, during a first PTAM. After six months, participants attended a second PTAM to reflect on the implementation of AAC. Participants were then followed for another three months. See Fig. 2 for an overview of the study timeline.

### Participants and recruitment

Eligible participants were employed in general practice as a GP, PN or doctor’s assistant (DA). We recruited PTAM groups which consisted of GPs and pharmacists, and asked the GPs to invite their PN and/or DA to enrol in the study as well. Different recruitment channels were used: newsletters directed at PTAM groups (through the Dutch Institute for Rational Use of Medicine, i.e., the organization which facilitates PTAM groups), newsletters of professional associations, e-mails sent directly to care groups throughout the Netherlands (in Dutch ‘zorggroepen’; these are management organisations which coordinate chain-based care for chronically ill patients), e-mails sent directly to contact persons of PTAM groups which participated in earlier research projects of the Dutch Institute for Rational Use of Medicine, e-mails sent directly to GPs working within two regions via two primary care research networks, and e-mails sent directly to practitioners who participated in an earlier study on implementation of smoking cessation care [7].

### Procedure and data collection

Participants received information on the study procedure, data protection and the anonymisation of research data. Subsequently, written informed consent was obtained from each participant before inclusion in the study. Participation was completely voluntary; participants were allowed to withdraw from the study at any time. During the study several variables were measured, of which those included in the current analyses are described below.

#### Main outcomes

Participants were asked to keep track of how many patients consulted them, how many patients they asked about smoking, how many smokers they advised to quit smoking, and how many smokers they referred to smoking cessation counselling. This data was collected during one week each month for the total duration of the study (resulting in nine timepoints T1-T9). The numbers were recorded in paper booklets. With regard to referrals, we also asked participants to note how they referred patients and whom they referred patients to. At the end of each data collection week, participants received an online questionnaire in which they could report their numbers and notes based on the booklet.

#### Baseline characteristics and evaluation

Participants also received additional online questionnaires: (i) a questionnaire at baseline to assess participant characteristics (e.g., age, profession, smoking status) and characteristics related to practice (e.g., socioeconomic position of patients, type of smoking cessation counselling offered in practice, number of referral options and interest in additional referral options, influence of COVID-19 on smoking cessation care); (ii) a questionnaire at the end of the study to evaluate AAC and assess effects of study participation on implementation of smoking cessation care (e.g., “As a result of this study I make sure to ask patients without smoking-related complaints about smoking”).

At the end of the study, participants received €50. We also distributed €500 (3x) and €1000 (1x) among those who completed all questionnaires.

### Statistical analysis

Statistical analysis was performed using IBM SPSS v27. Based on the self-reported data of the participants, we calculated for each timepoint (T1-T9) the proportion of patients that were asked about smoking (‘Ask’) and the proportion of patients that were advised to quit (‘Advise’). The changes in the proportions of ‘Ask’ and ‘Advise’ over time were modelled using linear mixed effects models. Model 1 included time (T1-T9) and intervention (pre-post) as fixed effects, and individual participants and PTAM groups as random effects. Model 2 additionally included an interaction term between time and intervention, and profession (GP vs. PN/DA) and negative influence of COVID-19 at baseline (no vs. yes) as fixed effects. We only included participants with data on at least one timepoint before the intervention (T1-T3) and at least one timepoint after the intervention (T4-T9).

We conducted a descriptive analysis with regard to referrals to smoking cessation counselling, because the numbers were too small to conduct a linear mixed effects analysis. We first determined, based on the self-reported data, whether participants had passively or proactively referred their patients at each timepoint, and also whether participants had referred patients internally or externally (i.e., inside or outside the practice). We then calculated for each participant which part of their referred patients (i.e., none/minority/half/majority/all) had been referred proactively (vs. passively) and externally (vs. internally) before (T1-T3) and after (T4-T9) introduction of the intervention.

Using the final questionnaire, we also conducted a descriptive analysis with regard to self-reported effects of study participation on implementation of smoking cessation care.

## Results

Ten PTAM groups with a total of 106 participants were included in the study. Participant characteristics are presented in Table 2. Most participants were female (81.9%), non-smoker (98.1%), and worked as a GP (60.0%). A small majority had previously received training in smoking cessation care (56.2%). All participants worked in a general practice which offered smoking cessation counselling, mostly individual counselling (99.0%) and telephone counselling (95.2%). At baseline, the majority of participants indicated that they would appreciate to have an additional referral option to smoking cessation counselling offered outside their practice (77.1%). At baseline, 40.0% reported that COVID-19 negatively influenced smoking cessation care within their practice.

**Table 2:** Characteristics of the participants and their general practice at baseline (*N* = 105)^a^

Variable	Category	n (%) / mean (SD)
Age		45.3 (9.2)
Gender	Male	19 (18.1)
	Female	86 (81.9)
Profession	General practitioner	63 (60.0)
	Practice nurse	36 (34.3)
	Doctor’s assistant	6 (5.7)
Smoking status	Smoker	2 (1.9)
	Non-smoker	103 (98.1)
Type of practice	Solo practice	17 (16.2)
	Duo practice	37 (35.2)
	Group practice	51 (48.6)
Socioeconomic position of patients	Mostly low	6 (5.7)
	Mostly middle	36 (34.3)
	Mostly high	4 (3.8)
	Mixed	52 (49.5)
	Don’t know	7 (6.7)
Received training in smoking cessation care	Yes	59 (56.2)
	No	46 (43.8)
Uses smoking cessation guideline with smokers	Never	44 (41.9)
	Sometimes	33 (31.4)
	Often	19 (18.1)
	(Almost) always	9 (8.6)
Attention in practice for smoking cessation	Almost no attention	3 (2.9)
	Some attention	58 (55.2)
	A lot of attention	44 (41.9)
Type of smoking cessation counselling offered within practice (multiple answers possible)	Individual counselling	104 (99.0)
	Group counselling	16 (15.2)
	Telephone counselling	100 (95.2)
Number of referral options for smoking cessation counselling^b^		2.0 (1.2)
Would appreciate additional referral option outside practice for smoking cessation counselling	Yes	81 (77.1)
	No	24 (22.9)
Smoking cessation care negatively influenced by COVID-19^c^	Yes	42 (40.0)
	No	63 (60.0)

^a^While 106 participants were included in the study, one participant did not complete the baseline questionnaire and therefore only the characteristics of 105 participants are presented here

^b^One participant who reported ‘99’ referral options was excluded

^c^We asked participants to describe the influence of COVID-19 on smoking cessation care, and categorised their answers into ‘negative influence’ versus ‘other’ (i.e., ‘positive/mixed/no/unclear influence’)

### Ask and advise

A total of 83 participants were included in the linear mixed effects models, as 23 participants did not report enough data to be included in the analyses. The group that was excluded from the analyses consisted of more men, GPs (vs. PN/DA) and smokers (vs. non-smokers) compared to the group that was included in the analyses, as shown in Supplementary File 1.

**Fig. 3:**
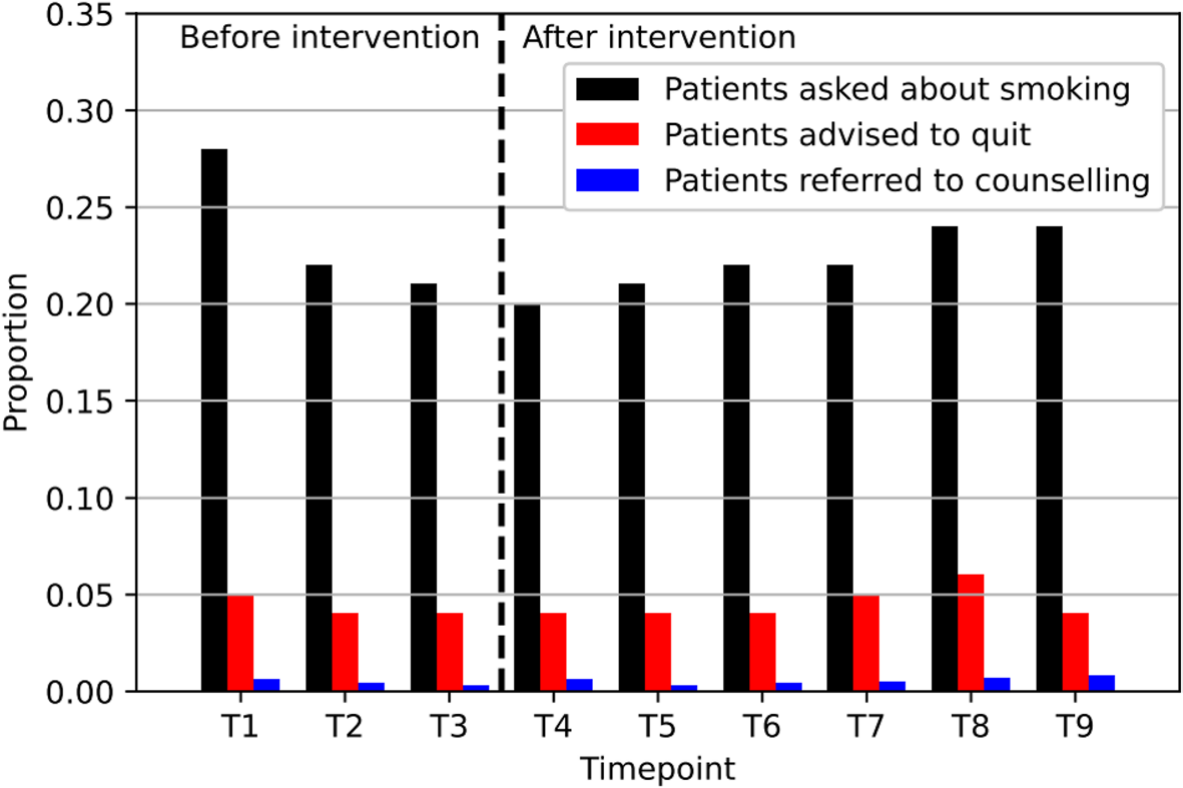
Unadjusted proportions over time of patients asked about smoking, advised to quit, and referred to behavioural counselling (*n*=83)

The 83 included participants provided consultations to a total of 29,112 patients (both smokers and non-smokers) during the entire study (10,427 patients before intervention, and 18,685 patients after intervention). Figure 3 shows the unadjusted proportions over time of patients asked about smoking, advised to quit, and referred to behavioural counselling. Most patients were asked about smoking at timepoint T1, and advised to quit smoking at timepoint T8. Results of the linear mixed effects models are presented in Table 3. The results of the fully adjusted model show that the proportion of patients that were asked about smoking (‘Ask’) significantly decreased with 0.049 (equivalent to roughly 5%) per timepoint between T1 and T3 (*p* < 0.001). The significant interaction effect between ‘Time’ and ‘Intervention’ shows that ‘Ask’ did not further decrease after T4, but slightly increased again with a difference of 0.005 (equivalent to 0.5%) per timepoint between T4 and T9 (*p* < 0.001). With regard to ‘Advise’ we found no significant change over time in both models.

**Table 3:** Results of the linear mixed effects models (*N* = 83)

**Model components**	***Ask***				***Advise***			
	***Model 1***		***Model 2***		***Model 1***		***Model 2***	
	**Estimate**	**SE**	**Estimate**	**SE**	**Estimate**	**SE**	**Estimate**	**SE**
Intercept	0.338*	0.041	0.063	0.126	0.061*	0.009	0.006	0.030
Time (T1-T9)	-0.002	0.004	-0.049*	0.010	0.002	0.002	-0.001	0.006
Intervention (pre vs. post)	-0.011	0.020	-0.148*	0.035	-0.013	0.010	-0.021	0.019
Time x Intervention			0.054*	0.011			0.003	0.006
Profession (GP vs. PN/DA)			0.288*	0.052			0.055*	0.011
Negative influence COVID-19 at baseline (no vs. yes)			-0.038	0.054			-0.013	0.012

^*^ *p* < 0.001

### Referrals

During the entire study, 41 participants referred a total of 147 patients to smoking cessation counselling. Descriptive statistics suggested that more proactive (vs. passive) referrals and more external (vs. internal) referrals took place after the intervention was introduced. Specifically, before the intervention 63.2% of participants proactively referred all interested patients to counselling. After the intervention, 76.7% of participants proactively referred their patients to counselling: 60.0% referred all patients proactively; 16.7% referred the minority, half or majority of their patients proactively. Also, before the intervention 13.6% of the participants referred their patients to counselling outside the practice (9.1% referred all patients externally, 4.5% referred half of their patients externally); this was 41.2% after the intervention (26.5% referred all patients externally, 2.9% referred a minority of their patients externally, 11.8% referred a majority of their patients externally).

### Other effects

A total of 65 participants completed the final questionnaire. Table 4 shows that the majority of these participants reported effects of study participation on the implementation of smoking cessation care. Participants mostly reported that the study convinced them of the added value of proactive referral of smokers (78.5%) and that they now know what the regional and/or national possibilities are for smoking cessation counselling (70.8%). These effects seemed more pronounced among GPs compared to PNs/DAs.

**Table 4:** Self-reported effects of study participation on implementation of smoking cessation care based on the last questionnaire (*n* = 65)

Effect	Yes – n (%)			No, and this was also not the case before participating in this study – n (%)			No, but this was already the case before participating in this study – n (%)		
“As a result of this study I…	Total	GP	PN/DA	Total	GP	PN/DA	Total	GP	PN/DA
…make sure to ask patients without smoking-related complaints about smoking.”	33 *(****50.8****)*	19 *(****57.6****)*	14 (43.8)	13 (20.0)	8 (24.2)	5 (15.6)	19 (29.2)	6 (18.2)	13 (40.6)
…make sure to give smokers a quit advice regardless of their motivation.”	40 *(****61.5****)*	24 *(****72.7****)*	16 *(****50.0****)*	7 (10.8)	3 (9.1)	4 (12.5)	18 (27.7)	6 (18.2)	12 (37.5)
…make sure to mention in the quit advice that counselling is the best way to quit smoking.”	42 *(****64.6****)*	24 *(****72.7****)*	18 *(****56.3****)*	6 (9.2)	2 (6.1)	4 (12.5)	17 (26.2)	7 (21.2)	10 (31.3)
…make sure to discuss different types of behavioural counselling with patients who want to quit smoking.”	33 *(****50.8****)*	16 (48.5)	17 *(****51.3****)*	12 (18.5)	9 (27.3)	3 (9.4)	20 (30.8)	8 (24.2)	12 (37.5)
…know what the regional and/or national possibilities are for smoking cessation counselling.”	46 *(****70.8****)*	24 *(****72.7****)*	22 *(****68.8****)*	7 (10.8)	3 (9.1)	4 (12.5)	12 (18.5)	6 (18.2)	6 (18.8)
…am convinced of the added value of proactive referral of smokers.”	51 *(****78.5****)*	26 *(****78.8****)*	25 *(****78.1****)*	5 (7.7)	3 (9.1)	2 (6.3)	9 (13.8)	4 (12.1)	5 (15.6)

Data were collected among 33 GPs and 32 PNs/DAs. Percentages over 50% are printed in bold

## Discussion

### Main findings

To our knowledge, this was the first study that investigated the influence of a comprehensive implementation strategy on the delivery of AAC within general practice during the COVID-19 pandemic. During the entire study, consultations were provided to 29,112 patients by 83 participants. The findings of this study show that the implementation strategy resulted in more patients being asked about smoking (‘Ask’). We observed an increase in the proportion of participants that proactively and externally referred their patients during the intervention period. Participants also reported positive effects of participating in the study, such as improved knowledge of the possibilities for smoking cessation counselling. The implementation strategy did not result in more patients being advised to quit smoking (‘Advise’).

### Interpretation of the findings

Our AAC implementation strategy consisted of different components, of which the main components were the two PTAMs in which participants were educated about the AAC method, made agreements on the implementation of AAC and reflected on these agreements. Previous research found that educational programs can be effective in helping primary care providers to identify smokers and offer advice and support [21]. Educational programs are especially effective when they actively engage primary care providers with the information they receive by providing a support tool, such as a physical card with information or an online toolkit, which we also provided to our participants [22]. A study conducted among Dutch GPs also found that formulating an action plan which states when, how, and by whom patients will be asked about smoking positively influenced GPs’ asking patients about smoking [23].

Our study shows that the implementation strategy was successful in two ways. First, we found that the proportion of participants that proactively referred a part of their patients increased with 13.5% after the intervention. Assuming that 17.6 times more proactively referred patients enrol in treatment compared to passively referred patients [18], our implementation strategy translated into roughly 5% more patients who enrolled into counselling during the COVID-19 pandemic. Considering the challenges faced by general practices during the COVID-19 pandemic [25], it is a positive finding that more participants were able to proactively refer a part of their patients. It is, however, important to note that the estimated impact would have been much greater (i.e., around 20% more patients enrolled into counselling) if participants had proactively referred all of their patients. Future implementation efforts should focus on increasing the proportion of patients that are proactively referred, for example by including prompts in the electronic health record or by providing performance feedback reports. Second, our results show that participants more often referred their patients to an external counsellor as a result of our implementation strategy. These are positive findings as most participants indicated that they would appreciate an extra referral option for patients who want to quit smoking. Especially during times in which general practices are faced with a high workload, being able to refer patients to an external counsellor ensures that patients receive cessation support while relieving the burden on healthcare providers within primary care.

Only two other studies have previously assessed the impact of an implementation strategy on the provision of AAC. One study conducted in primary care found that a comprehensive AAC implementation strategy consisting of training, performance feedback reports and the incorporation of an e-referral functionality in the electronic health record, resulted in more patients being asked about smoking and more smokers being advised to quit and connected to cessation support [26]. However, another study conducted in a Dutch university hospital found that an AAC implementation strategy consisting of education and reminders through text messages did not result in more patients being asked about smoking or more smokers being connected to a smoking cessation program [27]. According to the researchers the lack of an effect could be explained by other priorities and time pressure on the healthcare providers [27].

Considering the evidence in the literature, it is surprising that our comprehensive implementation strategy had a small positive effect on ‘Ask’ and no significant effect on ‘Advise’. Notably, most patients were asked about smoking at the beginning of the study, indicating that study participation may have been an intervention in itself. Although the proportion of ‘Ask’ sharply declined after timepoint T1, and significantly increased again after the implementation strategy was introduced, the level of ‘Ask’ displayed at timepoint T1 was not achieved again later in the study.

There may be several explanations for the modest effects we found. First, even though the need for providing smoking cessation support increased during the pandemic due to the fact that smokers face worse outcomes once infected with COVID-19 [28], we noticed that the COVID-19 pandemic adversely influenced the provision of smoking cessation care by our participants. In the questionnaires as well as the PTAMs, participants indicated that it was more difficult to discuss smoking with patients due to the telephone/online consultations and shifted priorities resulting from the COVID-19 pandemic. Also, several participants indicated that they experienced difficulty in staying engaged with the study as they did not have enough time to record notes in the paper booklet. Second, the desk card we provided to physically remind participants of AAC may not have been sufficient, as desk cards may be easily discarded. Reminders built into the electronic health record may be necessary to enhance the implementation of AAC in general practice. Third, several participants indicated during the second PTAM that most patients are not yet sure about quitting smoking, and as such cannot directly be referred to counselling. These patients are often first referred to the PN for one or more motivational conversations, and are later on referred to counselling once they are motivated to quit. Therefore, the low number of referrals which we found may be an underestimation. And finally, many participants, especially PNs, already quite actively provided smoking cessation care before participating in the study. Several participants indicated in the PTAMs that they already knew the smoking status of many of their patients or had already provided a quit advice in the previous year, and therefore did not bring up the subject again. Also, the descriptive results showed that the majority of participants already proactively referred their patients before the intervention. As such, selection bias in our sample of participants likely limited the extent to which improvements could be made in the delivery of AAC. We assume that, following nationwide rollout of the intervention, larger effects will be found among primary care providers who are less actively involved in providing smoking cessation care. We, however, also expect such primary care providers to be less inclined to receive the intervention in their PTAM groups. Thus, additional efforts may be needed to motivate primary care providers to address smoking cessation care in their PTAM groups.

### Limitations

A few limitations of this study must be addressed. First, it was not possible to extract the data from the electronic health record since our variables of interest are not routinely recorded in the system. As such, findings are based on self-report. It is possible that the recording of notes in the paper booklet may have made participants more aware of the care they provide and may have thus resulted in them more often providing smoking cessation care (Hawthorne effect) [29]. However, in view of the stressful conditions under which primary care providers worked during the COVID-19 pandemic [25], it is also likely that participants forgot or did not have enough time to record how often they asked patients about tobacco use, advised smokers to quit and referred smokers to counselling. We are therefore unsure whether data reported by the participants truly reflects what took place during a patient’s visit. However, this potential bias is likely to be the same before and after the intervention, such that results for differences should not be affected. Second, we could not determine the proportion of smokers that received a quit advice, because that would require knowing the smoking status of all patients, which typically is not the case in Dutch primary care. Therefore we could only compare proportions of all patients that received a quit advice before and after the intervention, which is sufficient to determine whether ‘Advise’ changed over time (assuming that the smoking prevalence did not change over time). Third, we were unable to statistically compare the proportion of referrals before and after intervention as the numbers of referrals were too low. Ideally, data should have been collected during the entire study. However, this was not possible as the burden of data collection would have been too high for many participants resulting in higher attrition rates. Fourth, although we collected data over nine months, we could not assess the sustainability of the intervention in the long term. This should be the topic of further research. Finally, we encountered difficulty in recruiting participants during the COVID-19 outbreak. We initially planned on conducting a stepped wedge cluster randomized trial, but were unable to recruit enough participants and therefore had to resort to a pre-post design which is associated with lower internal validity. On the other hand, switching to a simpler and more flexible design contributed to the feasibility of the study and thus the generalizability of the findings.

## Conclusions

Our findings indicate that a comprehensive implementation strategy can support primary care providers in offering smoking cessation care to patients, even under stressful COVID-19 conditions. The implementation strategy has the potential to increase the number of primary care providers who proactively refer patients to cessation counselling, which in turn would result in more smokers enrolling into treatment and ultimately quitting smoking. Additional implementation efforts are needed to increase the proportion of patients who receive a quit advice and proactive referral, for example by embedding reminders in the electronic health record. Further research should be undertaken to determine what is needed to sustain the implementation of AAC in the long term.

## Supplementary Information

**Additional file 1:** **Supplementary Table 1.** Differences in baseline characteristics between participants included in the analysis and participants not included in the analysis.

## Data Availability

The datasets generated and analysed during the current study are not publicly available, but are available from the corresponding author on reasonable request.

## Abbreviations

AAC: Ask-Advise-Connect
AAR: Ask-Advise-Refer
DA: Doctor’s assistant
GP: General practitioner
PN: Practice nurse
PTAM: Pharmaceutical Therapeutic Audit Meeting
