# Supplementary material for: The delivery of Ask-Advise-Connect for smoking cessation in Dutch general practice during the COVID-19 pandemic: results of a pre-post implementation study
Source: BMC Health Serv Res. 2023 Jun 19;23:654. doi: 10.1186/s12913-023-09692-1 (PMC10280870; doi:10.1186/s12913-023-09692-1)
Supplement: Supplementary file 1 — Additional file 1: Supplementary Table 1. Differences in baseline characteristics between participants included in the analysis and participants not included in the analysis. [file 12913_2023_9692_MOESM1_ESM.docx]

**Supplementary Table 1.** Differences in baseline characteristics between participants included in the analysis and participants not included in the analysis.

| **Variable** | **Category** | **Included in analysis (n=83)** | **Not included in analysis (n=22)^a^** |
| --- | --- | --- | --- |
| Age |  | 45.4 (9.1) | 45.0 (9.9) |
| Gender | Male | 8 (9.6) | 11 (50.0)* |
|  | Female | 75 (90.4) | 11 (50.0) |
| Profession | General practitioner | 45 (54.2) | 18 (81.8)* |
|  | Practice nurse | 35 (42.2) | 1 (4.5) |
|  | Doctor’s assistant | 3 (3.6) | 3 (13.6) |
| Smoking status | Smoker | 0 (0.0) | 2 (9.1)* |
|  | Non-smoker | 83 (100.0) | 20 (90.9) |
| Type of practice | Solo practice | 12 (14.5) | 5 (22.7) |
|  | Duo practice | 30 (36.1) | 7 (31.8) |
|  | Group practice | 41 (49.4) | 10 (45.5) |
| Socioeconomic status of patients | Mostly low | 5 (6.0) | 1 (4.5) |
|  | Mostly middle | 28 (33.7) | 8 (36.4) |
|  | Mostly high | 4 (4.8) | 0 (0.0) |
|  | Mixed | 40 (48.2) | 12 (54.5) |
|  | Don’t know | 6 (7.2) | 1 (4.5) |
| SCC training | Yes | 50 (60.2) | 9 (40.9) |
|  | No | 33 (39.8) | 13 (59.1) |
| Use of SCC guideline with smokers | Never | 32 (38.6) | 12 (54.5) |
|  | Sometimes | 25 (30.1) | 8 (36.4) |
|  | Often | 17 (20.5) | 2 (9.1) |
|  | (Almost) always | 9 (10.8) | 0 (0.0) |
| Attention in practice for smoking cessation | Almost no attention | 2 (2.4) | 1 (4.5) |
|  | Some attention | 45 (54.2) | 13 (59.1) |
|  | A lot of attention | 36 (43.4) | 8 (36.4) |
| Type of smoking cessation counselling offered within practice | Individual counselling | 83 (100.0) | 21 (95.5) |
|  | Group counselling | 12 (14.5) | 4 (18.2) |
|  | Telephone counselling | 80 (96.4) | 20 (90.9) |
| Number of referral options for smoking cessation counselling |  | 2.1 (1.1) | 1.8 (1.5) |
| Would appreciate additional referral option outside practice for smoking cessation counselling | Yes | 66 (79.5) | 15 (68.2) |
|  | No | 17 (20.5) | 7 (31.8) |

^a^ Officially 23 participants were excluded from the linear mixed effects models, but one participant did not complete the baseline questionnaire and therefore only the characteristics of 22 participants are presented here.

*Chi-square test showed significant difference (*p*<0.05).
